# Racial and ethnic variation in the prevalence of knee osteoarthritis among older U.S. adults, including Asian ethnic populations

**DOI:** 10.1016/j.ocarto.2026.100761

**Published:** 2026-02-24

**Authors:** Julia G. Costantini, Jeanne A. Darbinian, Elisha A. Garcia, Malini Chandra, Joan C. Lo

**Affiliations:** a Department of Medicine, Kaiser Permanente Oakland Medical Center, Oakland, CA, USA; b Division of Research, Kaiser Permanente Northern California, Pleasanton, CA, USA; c Department of Health Systems Science, Kaiser Permanente Bernard J. Tyson School of Medicine, Pasadena, CA, USA

**Keywords:** Osteoarthritis, Knee, Asian, Chinese, Filipino, South Asian

## Abstract

**Objective:**

National surveys suggest that US Asian adults have lower prevalence of osteoarthritis compared to White and Black adults, but disaggregated subgroup data are limited. This study examined the burden of knee osteoarthritis among older US adults by race and ethnicity, including Asian subgroup differences.

**Design:**

Data from a large integrated healthcare delivery system in northern California were used to identify adults aged 50–89 years during 2017–2019 who had body mass index (BMI) measured. Knee osteoarthritis was defined by ≥ 2 diagnoses (ICD-10 M17.x) during 2017–2019. The association of race/ethnicity and prevalence of knee osteoarthritis was examined using modified Poisson regression, adjusting for age and BMI level.

**Results:**

Among 1,183,576 adults (mean age 64.3 ± 9.6 years; 54.3 % female; 54.7 % White, 17.8 % Asian/Pacific Islander, 11.9 % Hispanic, and 6.7 % Black), 101,959 (8.6 %) had diagnosed knee osteoarthritis. For males, prevalence of knee osteoarthritis was higher for South Asian (adjusted prevalence ratio, aPR 1.51), modestly higher for Black (aPR 1.16), and similar or lower for Vietnamese, Chinese, Japanese, Filipino, Hispanic, and Native Hawaiian/Pacific Islander (NHPI) (aPRs 0.70–1.05) versus White males. For females, prevalence was higher for South Asian (aPR 2.15), modestly or somewhat higher for Hispanic, Filipina, Vietnamese, and Black (aPRs 1.14–1.32), and similar or lower for Japanese, Chinese, and NHPI (aPRs 0.77–1.05) versus White females.

**Conclusions:**

Knee osteoarthritis prevalence varied substantially by race/ethnicity with marked differences among Asian subgroups. Notably, prevalence was 1.5-to-2-fold higher for South Asian compared to White adults, surpassing Black adults. Delineation of reasons underlying higher knee osteoarthritis burden among high-risk ethnic populations is needed.

## 1 Introduction

Osteoarthritis affects up to 33 million adults in the US [1], and its prevalence is projected to continue rising [2], attributable in part to a growing population of older adults with increased life expectancy and obesity [3,4]. The knee is the most commonly affected joint for osteoarthritis [2,3,5], where older age, female sex, obesity, and prior joint injury are known risk factors [3,6,7]. Despite its substantial public health burden, the epidemiology of osteoarthritis among US populations has been primarily characterized among non-Hispanic White, Black, and Hispanic populations [4,8,9], whereas more limited data exist for Asian as well as Native Hawaiian and Pacific Islander (NHPI) populations. Current reports based on nationally representative US data suggest that Asian adults have lower prevalence of arthritis or osteoarthritis compared to non-Hispanic White and Black adults [1,[10], [11], [12]]. The National Center for Health Statistics also reported a much lower prevalence of arthritis among the Asian population (11.4 %) compared to the general US population (21.1 %), but a similar prevalence among the NHPI population (19.7 %) [12]. However, among Hawaiian participants in the Behavioral Risk Factor Surveillance Survey, NHPI males were diagnosed with arthritis at a younger age and those with obesity had a much higher prevalence of arthritis than White or Asian males with obesity [13]. While these findings were not restricted to osteoarthritis, the most common subtype among diagnosed arthritis conditions [1], they point to population differences independent of weight status.

On a global level, large increases in the prevalence of osteoarthritis have been observed in East Asian and South Asian countries between 1990 and 2019 [5]. However, studies of osteoarthritis within the US have rarely disaggregated data by Asian subpopulations (e.g., Chinese, Filipino, Japanese, Korean, South Asian, and Vietnamese) despite well-documented variation in chronic disease and health outcomes across these groups [14]. To address this knowledge gap, we examined an ethnically diverse community-based population of US older adults receiving healthcare to characterize the clinical burden of knee osteoarthritis by race and ethnicity, including among disaggregated Asian subgroups.

## 2 Methods

This cross-sectional study used electronic health record (EHR) data from Kaiser Permanente Northern California (KPNC) and was approved by the KPNC Institutional Review Board with a waiver of informed consent.

### 2.1 Study population

The study cohort included male and female adults aged 50–89 years (defined as age on July 1, 2018) who were KPNC health plan members during the three-year time period, 2017–2019 (at least nine months of KPNC membership in each year), and had height and weight measured at an outpatient clinic encounter during 2016–2020.

### 2.2 Outcome and covariates

Knee osteoarthritis was defined by having ≥2 diagnoses (ICD-10 M17.x) during 2017–2019 from hospital, emergency, outpatient, and institutional stay clinical encounters. Race and ethnicity were classified based on self-reported information from EHR or administrative data sources. Body mass index (BMI), calculated from weight and height, was categorized as <25.0 kg/m^2^, 25.0 to <30.0 kg/m^2^ (standard cut-point for overweight), 30.0 to <35.0 kg/m^2^ (standard cut-point for class 1 obesity), 35.0 to <40.0 kg/m^2^ (standard cut-point for class 2 obesity), and ≥40.0 kg/m^2^ (standard cut-point for class 3 obesity) using the BMI measurement closest to July 1, 2018. A total of 76.4 % had BMI ascertained in 2018, and 96.8 % with BMI had measurements during 2017–2019; only 5.1 % were missing BMI and were not included in this study. Because lower Asian-specific BMI cut-points have been proposed as intervention thresholds for Asian adults, a population known to have a higher burden of cardiometabolic health conditions at lower BMI levels, we also examined results when using lower proposed intervention cut-points of <23.0 kg/m^2^, 23.0 to <27.5 kg/m^2^, 27.5 to <32.5 kg/m^2^, 32.5 to <37.5 kg/m^2^, and ≥37.5 kg/m^2^ for Asian adults [15].

### 2.3 Statistical analyses

The prevalence of knee osteoarthritis was age-adjusted using population weights from the US Census 2018 American Community Survey data (adults aged 50–89 years in 5-year age strata) and reported separately for male and female populations. The association of race and ethnicity with knee osteoarthritis prevalence was examined using modified Poisson regression models, adjusting for age and BMI category. Adjusted prevalence ratios (aPR) are reported with 95 % confidence intervals [CI]. Analyses were conducted using SAS statistical software v9.4 (SAS Institute, Cary, NC). A two-sided p-value of <0.05 was considered significant.

## 3 Results

Among 1,183,576 adults, 54.3 % were female and 44.9 % were aged 65 years and older. The mean age was 64.3 ± 9.6 years. The cohort was racially and ethnically diverse, with 647,814 (54.7 %) White, 140,821 (11.9 %) Hispanic, 79,157 (6.7 %) Black, 210,709 (17.8 %) Asian or NHPI, and 105,075 (8.9 %) of other or unknown (0.8 %) race. The Asian/NHPI population included 59,009 (28.0 %) Filipino, 53,911 (25.6 %) Chinese, 14,729 (7.0 %) South Asian, 10,368 (4.9 %) Japanese, 9441 (4.5 %) Vietnamese, 6355 (3.0 %) NHPI, and 56,896 (27.0 %) with unspecified, other, or mixed Asian/NHPI ethnicity.

About one-third of all male (35.0 %) and female (34.0 %) adults had BMI ≥30 kg/m^2^ (i.e., obesity), but obesity varied substantially by race and ethnicity (Table 1, Table 2). For males, the proportion with obesity ranged from 46 % (Hispanic and Black), 41 % (NHPI), 37 % (White), 20–22 % (Japanese and Filipino), 18 % (South Asian), 9 % (Chinese), and 6 % (Vietnamese) using the standard BMI threshold for obesity, but ranged higher at 38–44 % (South Asian, Japanese, and Filipino) and 18–23 % (Vietnamese and Chinese) when the Asian-specific threshold of BMI ≥27.5 kg/m^2^ was used. For females, obesity ranged from 57 % (Black), 46 % (Hispanic), 43 % (NHPI), 35 % (White), 24 % (South Asian), 18 % (Filipina), 13 % (Japanese), 7 % (Chinese), and 5 % (Vietnamese) using the standard BMI threshold for obesity, and higher at 42 % (South Asian), 34 % (Filipina), 23 % (Japanese), and 13–15 % (Vietnamese and Chinese) using the Asian-specific threshold.

**Table 1:** Age, weight classification, and knee osteoarthritis by sex and major race/ethnicity groups.

MALE	White	Hispanic	Black	Asian/NHPI	Other/Unknown
	N = 299,053	N = 67,576	N = 33,668	N = 94,919	N = 46,198
Mean age	65.0 ± 9.5	61.4 ± 9.2	63.0 ± 9.2	63.1 ± 9.3	63.4 ± 9.6
Age group (years)					
50-64	51.3 %	68.5 %	61.1 %	60.0 %	59.7 %
65-74	30.9 %	20.5 %	25.6 %	26.8 %	25.5 %
75-89	17.8 %	11.0 %	13.3 %	13.2 %	14.8 %
BMI (standard cut-points)					
<25.0 kg/m^2^	20.5 %	12.3 %	17.4 %	39.5 %	19.8 %
25.0 to <30.0	42.4 %	42.0 %	36.6 %	45.0 %	42.3 %
30.0 to <35.0	24.2 %	30.6 %	27.6 %	12.2 %	25.1 %
35.0 to <40.0	8.7 %	10.5 %	11.5 %	2.5 %	8.8 %
≥40.0	4.3 %	4.6 %	6.9 %	0.8 %	4.0 %
Knee osteoarthritis	7.8 %	7.2 %	8.7 %	4.5 %	7.2 %

FEMALE	White	Hispanic	Black	Asian/NHPI	Other/Unknown
	N = 348,761	N = 73,245	N = 45,489	N = 115,790	N = 58,877
Mean age	65.6 ± 9.7	62.3 ± 9.6	63.7 ± 9.5	63.3 ± 9.5	63.6 ± 9.8
Age group (years)					
50-64	48.7 %	64.6 %	57.8 %	59.6 %	58.8 %
65-74	31.5 %	22.0 %	26.9 %	26.5 %	25.2 %
75-89	19.8 %	13.3 %	15.3 %	14.0 %	16.0 %
BMI (standard cut-points)					
<25.0 kg/m^2^	34.8 %	19.1 %	15.6 %	54.4 %	29.7 %
25.0 to <30.0	30.7 %	34.5 %	27.6 %	32.0 %	32.7 %
30.0 to <35.0	18.7 %	26.0 %	26.4 %	10.0 %	20.8 %
35.0 to <40.0	9.2 %	12.5 %	16.1 %	2.6 %	9.9 %
≥40.0	6.7 %	8.0 %	14.3 %	1.0 %	7.0 %
Knee osteoarthritis	9.9 %	10.7 %	14.8 %	7.1 %	10.3 %

NHPI = Native Hawaiian or Pacific Islander; BMI = body mass index.

**Table 2:** Age, body mass index, and knee osteoarthritis by sex and Asian or NHPI subgroups^a^.

MALE	Chinese	Filipino	South Asian	Vietnamese	Japanese	NHPI
	N = 23,793	N = 24,089	N = 7905	N = 4742	N = 4052	N = 3235
Mean age	65.7 ± 9.7	63.7 ± 9.1	62.5 ± 9.2	62.2 ± 9.1	67.4 ± 9.8	60.6 ± 7.9
Age group (years)						
50-64	48.0 %	57.0 %	62.4 %	63.9 %	41.8 %	71.6 %
65-74	32.3 %	29.4 %	25.2 %	24.0 %	34.2 %	22.1 %
75-89	19.6 %	13.5 %	12.5 %	12.1 %	24.1 %	6.3 %
BMI (standard cut-points)						
<25.0 kg/m^2^	50.3 %	28.1 %	34.7 %	56.0 %	35.1 %	19.8 %
25.0 to <30.0	40.7 %	50.5 %	47.1 %	38.0 %	44.3 %	38.9 %
30.0 to <35.0	7.5 %	16.7 %	14.5 %	5.3 %	16.1 %	24.8 %
35.0 to <40.0	1.1 %	3.6 %	3.0 %	0.6 %	3.5 %	10.6 %
≥40.0	0.3 %	1.1 %	0.8 %	0.2 %	1.0 %	6.0 %
BMI (Asian cut-points)						
<23.0 kg/m^2^	25.6 %	11.3 %	15.5 %	27.9 %	16.0 %	- -
23.0 to <27.5	51.5 %	45.2 %	46.0 %	54.4 %	43.8 %	- -
27.5 to <32.5	19.2 %	33.4 %	30.5 %	15.4 %	30.7 %	- -
32.5 to <37.5	3.0 %	8.1 %	6.1 %	2.0 %	7.2 %	- -
≥37.5	0.6 %	2.1 %	1.8 %	0.3 %	2.3 %	- -
Knee osteoarthritis	4.3 %	5.3 %	8.4 %	3.2 %	5.7 %	6.6 %

FEMALE	Chinese	Filipina	South Asian	Vietnamese	Japanese	NHPI
	N = 30,118	N = 34,920	N = 6824	N = 4699	N = 6316	N = 3120
Mean age	64.4 ± 9.6	64.0 ± 9.5	62.0 ± 9.1	62.3 ± 8.9	68.4 ± 10.8	61.3 ± 8.6
Age group (years)						
50-64	53.9 %	56.2 %	65.0 %	62.8 %	38.8 %	69.3 %
65-74	29.8 %	28.4 %	23.7 %	25.9 %	30.4 %	21.5 %
75-89	16.3 %	15.3 %	11.4 %	11.3 %	30.8 %	9.2 %
BMI (standard cut-points)						
<25.0 kg/m^2^	68.5 %	42.3 %	34.6 %	71.7 %	60.4 %	23.5 %
25.0 to <30.0	24.9 %	39.4 %	41.8 %	23.6 %	26.9 %	33.3 %
30.0 to <35.0	5.3 %	13.7 %	17.3 %	3.8 %	8.9 %	22.5 %
35.0 to <40.0	1.0 %	3.5 %	4.7 %	0.7 %	2.7 %	12.1 %
≥40.0	0.4 %	1.1 %	1.6 %	0.2 %	1.1 %	8.6 %
BMI (Asian cut-points)						
<23.0 kg/m^2^	47.1 %	22.9 %	18.5 %	49.3 %	41.8 %	- -
23.0 to <27.5	38.3 %	42.9 %	39.6 %	38.1 %	35.4 %	- -
27.5 to <32.5	11.8 %	24.9 %	29.4 %	10.6 %	15.9 %	- -
32.5 to <37.5	2.2 %	7.1 %	9.4 %	1.6 %	4.8 %	- -
≥37.5	0.7 %	2.2 %	3.1 %	0.4 %	2.1 %	- -
Knee osteoarthritis	6.3 %	8.4 %	15.2 %	6.6 %	6.5 %	8.9 %

NHPI = Native Hawaiian or Pacific Islander; BMI = body mass index.

a Not included in this table are individuals of other, mixed, or unspecified Asian/NHPI ethnicity.

Overall, 101,959 (8.6 %) adults had diagnosed knee osteoarthritis during 2017–2019, with 38,662 (37.9 %) male and 63,297 (62.1 %) female. For both males and females, those with knee osteoarthritis were significantly more likely to be aged ≥65 years (63.4 % vs 42.2 % for male, 66.9 % vs 43.7 % for female, p < 0.001) and have obesity (49.7 % vs 33.9 % for male, 52.2 % vs 32.0 % for female, p < 0.001) compared with those who did not have diagnosed knee osteoarthritis. Males with knee osteoarthritis were also more likely to be White (60.3 % vs 54.9 %) and Black (7.6 % vs 6.1 %) and less likely to be Asian (11.0 % vs 18.0 %, p < 0.001 for all comparisons). Females with knee osteoarthritis were also more likely to be Black (10.6 % vs 6.7 %), slightly more likely to be Hispanic (12.3 % vs 11.3 %) and less likely to be Asian (12.9 % vs 18.6 %, p < 0.001 for all comparisons). As shown in Table 1, Table 2, the proportion with knee osteoarthritis varied across racial and ethnic groups.

**Fig. 1:**
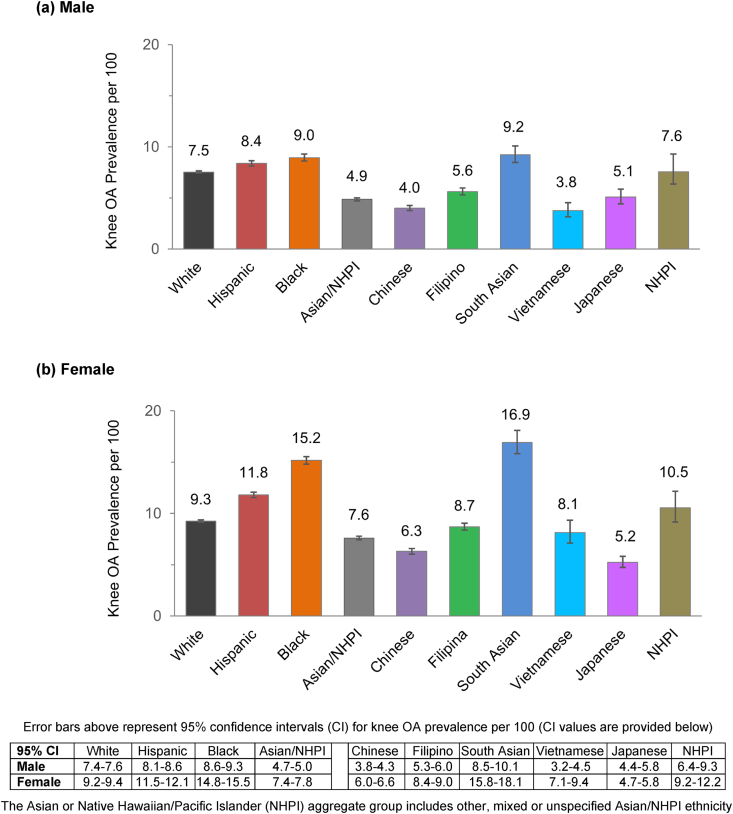
Age-adjusted prevalence of knee osteoarthritis (OA) among adults aged 50–89 years.

The age-adjusted prevalence (per 100) of diagnosed knee osteoarthritis was 7.3 [95 % CI 7.2–7.3] for male and 9.8 [9.7–9.8] for female adults, overall. Among males, prevalence was higher for Black (9.0) and Hispanic (8.4) compared to White males (7.5), and lower for Asian/NHPI males (4.9) when examined as an aggregate group (Fig. 1a). Females had a similar pattern – prevalence was higher for Black (15.2) and Hispanic (11.8) compared to White females (9.3), and lower for Asian/NHPI females (7.6) (Fig. 1b). Large variation in prevalence was also evident among the Asian/NHPI subgroups. For Asian/NHPI males, the age-adjusted prevalence of knee osteoarthritis (per 100) was highest for South Asian (9.2) and NHPI (7.6) followed by Filipino (5.6) and Japanese (5.1) males, and lower for Chinese (4.0) and Vietnamese (3.8) males. Among Asian/NHPI females, the age-adjusted prevalence was highest for South Asian (16.9), followed by NHPI (10.5), Filipina (8.7), and Vietnamese (8.1) females and lower for Chinese (6.3) and Japanese (5.2) females. Furthermore, for each racial and ethnic group except Japanese adults, the age-adjusted prevalence of knee osteoarthritis was higher for females, especially among Black and South Asian females compared to their male counterparts.

**Fig. 2:**
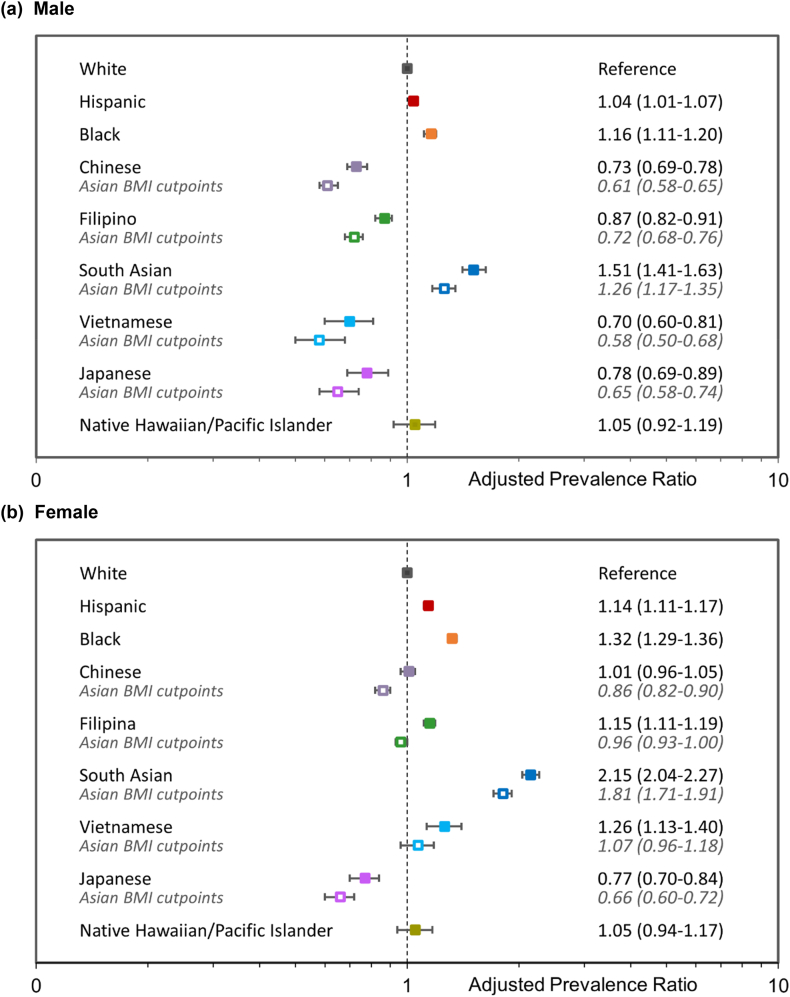
Prevalence ratios for knee osteoarthritis adjusted for age and body mass index category Modified Poisson regression models adjusted for age and body mass index (BMI) category (see methods) using standard BMI cut-points for all race/ethnicity groups and Asian-specific BMI cut-points for Asian groups (grey text).

Sex-specific adjusted prevalence ratios (aPR) that accounted for age and BMI level were also examined across demographic groups using modified Poisson regression (Fig. 2). Compared to White males, the adjusted prevalence of knee osteoarthritis (using standard BMI cut points) was higher for South Asian males (aPR 1.51), modestly higher for Black males (aPR 1.16), similar for Hispanic and NHPI males (aPRs 1.04–1.05) and lower for Vietnamese, Chinese, Japanese, and Filipino males (aPRs 0.70–0.87). When Asian-specific thresholds were used to categorize BMI level for Asian subgroups, the differences were even greater for Vietnamese, Chinese, Japanese, and Filipino males (aPRs 0.58–0.72) but attenuated for South Asian males (aPR 1.26) when compared to White males. For females, compared to their White counterparts, the adjusted prevalence of knee osteoarthritis (using standard BMI cut-points) was over twofold higher for South Asian females (aPR 2.15), modestly or somewhat higher for Hispanic, Filipina, Vietnamese, and Black females (aPRs 1.14–1.32); similar for Chinese and NHPI females (aPRs 1.01–1.05); and lower for Japanese females (aPR 0.77). When Asian-specific thresholds were used to categorize BMI level, Chinese, Filipina, and Vietnamese females had modestly lower or similar prevalence (aPR 0.86–1.07), Japanese had even lower prevalence (aPR 0.66) and South Asian females had nearly two-fold higher prevalence (aPR 1.81) compared to White females.

## 4 Discussion

This large and contemporary population study of nearly 1.2 million older US adults from a large integrated healthcare delivery system highlights the substantial variation in burden of diagnosed knee osteoarthritis across racial and ethnic groups, including Asian/NHPI subgroups. The prevalence of knee osteoarthritis was higher among female compared to male adults, consistent with prior epidemiologic and population-based studies where proposed factors contributing to these differences include anatomic/biomechanical factors, genetics, hormonal influences, obesity, and functional outcomes [7,16,17]. Furthermore, when compared to White adults, the sex-specific prevalence of knee osteoarthritis, adjusted for age and BMI level, was 1.2–1.3-fold higher for Black followed by Hispanic adults, with larger differences among females. Notably, our study identified an even higher adjusted prevalence of knee osteoarthritis among South Asian male (1.3–1.5-fold) and South Asian female adults (1.8–2.2-fold) in comparison to White adults, whereas Chinese, Japanese, Vietnamese, and Filipino populations had similar, lower, or only modestly higher adjusted prevalence (depending on sex and BMI criteria) and NHPI adults had similar adjusted prevalence. Future studies should examine the extent to which BMI or adiposity mediates population differences in knee osteoarthritis across demographic groups.

This is the largest study to date to examine knee osteoarthritis prevalence in a population of over 50,000 Chinese, nearly 60,000 Filipino, and nearly 15,000 South Asian adults residing in the US, in comparison to White, Black, and Hispanic populations within the same healthcare setting. Prior investigations have focused on smaller US regions, including a study of 6735 NHPI, White, and Asian adults in Hawaii [13], an early report from 780 Chinese, Cambodian, and Vietnamese adults in an urban Chicago community [18], and representative population samples to derive national prevalence estimates, the latter reporting on Asian as an aggregate group. Many contemporary studies have used recent data from population surveys such as the Behavioral Risk Factor Surveillance System [19], the National Health Interview Survey [20], and the National Health and Nutrition Examination Survey [1,11,21] which rely on self-reported diagnosis of arthritis or osteoarthritis, not necessarily localized to the knee.

The mechanism underlying the disproportionate burden of knee osteoarthritis among South Asian adults is unclear and may reflect a complex interplay of genetic predisposition, biology, cultural practices, occupational exposures, and access to care [6,22]. As one of the fastest growing Asian origin groups within the US [23], this ethnic population remains extremely understudied and includes a large immigrant population. Our results highlight the considerable heterogeneity among US Asian populations which in aggregate have been considered to have much lower arthritis prevalence than the general US population [12] and White and Black adults [1,10,11,19]. More globally, the burden of osteoarthritis has risen among countries in East, South, and Southeast Asia [5,24,25]. Collectively, these findings emphasize the importance of examining disaggregated US Asian populations and the need for more research to better understand potential osteoarthritis risk pathways within South Asian and other higher risk populations that may differ from those in other racial or ethnic groups [8].

This study has several limitations. First, osteoarthritis of the knee was identified from diagnosis codes rather than through systematic clinical or radiographic assessment. Thus, undiagnosed cases were missed and disease severity could not be ascertained. We also cannot exclude the possibility of misclassification, although case ascertainment required two diagnoses. Second, while major risk factors (sex, age, weight status) were accounted for in our analyses, information on other osteoarthritis risk factors (e.g., prior joint injury, activity level, and occupation) were not available [7]. Third, we acknowledge the limitation of BMI as a surrogate measure of adiposity and recognize the need for more accurate adiposity measures [26]. Fourth, our study was limited to a northern California population, and findings may not be generalizable the broader US population or other geographic regions. However, the demographic distribution of KPNC is comparable to the general population in the region [27]. Fifth, we report cross-sectional data from different Asian populations but recognize that nativity, immigration, employment, and related sociodemographic factors affect osteoarthritis risk [2], and geographic variation may exist. Finally, pain, disability, and treatment for knee osteoarthritis were not examined [28,29], and we acknowledge that differences in healthcare utilization among racial and ethnic groups could bias prevalence estimates. Overall, these emerging trends observed support the need for systematic examination and surveillance of knee osteoarthritis in understudied ethnic populations.

In summary, our study identified key demographic differences in the epidemiology of knee osteoarthritis among older US Asian adults in a large community-based healthcare population with similar access to care. To our knowledge, this study is the first to examine knee osteoarthritis prevalence among disaggregated Asian/NHPI subgroups in a large northern California population, the state where 29 % of all US Asians currently reside [30]. Among the five largest Asian subgroups examined, our findings document notable heterogeneity and a much higher risk among South Asian adults. However, further research is needed, especially among other Asian subgroups not yet examined [31]. A more nuanced understanding of subgroup-specific patterns may inform efforts to improve musculoskeletal health and physical function among diverse populations of older US adults.

## Author contributions

Joan Lo, Julia Costantini, Jeanne Darbinian, Malini Chandra, and Elisha Garcia contributed to study conception and/or development of study approach and methods; Malini Chandra contributed to cohort development; Jeanne Darbinian abstracted the data and conducted all data analyses; all authors provided input on the interpretation of data; Julia Costantini, Joan Lo, and Elisha Garcia drafted the initial manuscript, and all authors provided input and manuscript edits for important intellectual content. All authors approved the final manuscript for publication. Joan Lo obtained funding and provided supervision for the study.

## Funding source

This study was funded by the National Institute on Aging at the National Institutes of Health (R01 AG069992). The sponsor had no role in the study concept and design, acquisition of data, analyses, and interpretation of data, and writing of the manuscript. The content is solely the responsibility of the authors and does not represent the official views of the National Institutes of Health or Kaiser Permanente.

## Conflict of interest

Jeanne Darbinian, Elisha Garcia, Malini Chandra, and Joan Lo received funding from the National Institute on Aging related to this work but have no other conflicts of interest to report. Julia Costantini has no conflicts of interest to report.
